# Desert plant bacteria reveal host influence and beneficial plant growth properties

**DOI:** 10.1371/journal.pone.0208223

**Published:** 2018-12-12

**Authors:** Abdul Aziz Eida, Maren Ziegler, Feras F. Lafi, Craig T. Michell, Christian R. Voolstra, Heribert Hirt, Maged M. Saad

**Affiliations:** 1 Desert Agriculture Initiative, King Abdullah University of Science and Technology (KAUST), Biological and Environmental Sciences and Engineering Division (BESE), Thuwal, Kingdom of Saudi Arabia; 2 Red Sea Research Center, King Abdullah University of Science and Technology (KAUST), Biological and Environmental Sciences and Engineering Division (BESE), Thuwal, Kingdom of Saudi Arabia; Universita degli Studi del Piemonte Orientale Amedeo Avogadro, ITALY

## Abstract

Deserts, such as those found in Saudi Arabia, are one of the most hostile places for plant growth. However, desert plants are able to impact their surrounding microbial community and select beneficial microbes that promote their growth under these extreme conditions. In this study, we examined the soil, rhizosphere and endosphere bacterial communities of four native desert plants *Tribulus terrestris*, *Zygophyllum simplex*, *Panicum turgidum* and *Euphorbia granulata* from the Southwest (Jizan region), two of which were also found in the Midwest (Al Wahbah area) of Saudi Arabia. While the rhizosphere bacterial community mostly resembled that of the highly different surrounding soils, the endosphere composition was strongly correlated with its host plant phylogeny. In order to assess whether any of the native bacterial endophytes might have a role in plant growth under extreme conditions, we analyzed the properties of 116 cultured bacterial isolates that represent members of the phyla Proteobacteria, Bacteroidetes, Actinobacteria and Firmicutes. Our analysis shows that different strains have highly different biochemical properties with respect to nutrient acquisition, hormone production and growth under stress conditions. More importantly, eleven of the isolated strains could confer salinity stress tolerance to the experimental model plant *Arabidopsis thaliana* suggesting some of these plant-associated bacteria might be useful for improving crop desert agriculture.

## Introduction

According to the United Nations Organization, the current world population of 7.6 billion is expected to increase beyond 9.8 billion by the year 2050 [1]. The dramatically expanding human population is accompanied by environmentally destructive activities such as deforestation and the overuse of chemical fertilizers and pesticides in agriculture. Furthermore, global warming, as a consequence of greenhouse gas emissions, aggravates abiotic stresses and leads to reductions in cultivatable land and agricultural productivity [2–4]. Abiotic stresses, such as drought, salinity, extreme temperatures, UV radiation and nutrient deficiency and/or inaccessibility cause more than 50% of losses in major crop yield [5, 6]. As a consequence, the need to find environmentally friendly, cost-efficient and sustainable approaches to secure food availability for a growing population has become the subject of intense research [7].

Desert and arid regions cover about one quarter of the Earth’s land surface and encompass many of the challenges to increase agricultural productivity [8]. Deserts represent one of the harshest terrestrial ecosystems on Earth, and are characterized by high levels of solar radiation, low levels of rainfall and extreme temperatures. In addition, desert soils are characterized by low water retention, low nutrient levels and high salinity [9]. Although deserts seem to appear inhabitable to living organisms, a wide diversity of organisms, including plants, have adapted to these extreme conditions by evolving mechanisms to adjust to this environment. Desert plants developed several adaptation strategies, such as having deep and extensive root systems for exploiting the soil at great depths. Others, such as agaves and cacti, have a crassulacean acid metabolism (CAM) which allows plants to fix carbon dioxide during the night, thereby preventing high evaporation during the day [10–13]. Additionally, a key factor by which plants can adapt to these conditions is putatively their microbiome [14–17].

Microbes associated with the roots of desert plants are capable of promoting plant growth and stress tolerance in crop species [18–20]. Bacteria and fungi play crucial roles in nutrient cycling in desert ecosystems [21, 22] and are indispensable partners to plants [23, 24]. The use of beneficial bacteria, termed plant growth promoting bacteria (PGPB), as biofertilizers for improving plant growth is widely recognized [25–27]. Based on their colonizing strategy, PGPB can be rhizospheric (living in the rhizosphere, a thin layer surrounding roots), epiphytic (at the surface of roots or leaves) or endophytic (inside the plant body). PGPB can affect plant growth through direct or indirect mechanisms [28]. These include biocontrol mechanisms, such as the production of antimicrobial compounds against pathogenic bacteria or fungi, or inducers of systemic resistance against soil-borne pathogens. Furthermore, PGPB can help plants in the acquisition of nutrients, such as nitrogen fixation, phosphate and zinc solubilization and siderophore production for sequestering iron (Fe^3+^); or in the modulation of phytohormone levels, such as auxin, indole-acetic acid (IAA), cytokinins, gibberellins, or ethylene (lower the level of its precursor 1-aminocyclopropane-1-carboxylate, ACC) [28–30]. However, many of the mechanisms by which microbes, especially bacteria associated to desert plants, induce abiotic stress tolerance in plants are still poorly understood.

The aim of the study was to assess the properties of the bacterial communities associated with the soil, rhizosphere and endosphere of four pioneer desert plant species that were isolated from two locations in Saudi Arabia. For this purpose, Due to the ability of the host desert plants to live in sandy soil under poor nutrient conditions and osmotic stress, we hypothesized that the endosphere compartment would contain bacterial isolates which possess PGP traits that assist plants in nutrient acquisition and the ability to survive under these extreme conditions. Therefore, we generated a library of culturable bacterial strains isolated from the endosphere of the four desert plant species and characterized their commonly associated plant growth promotion properties. A selected number of these strains were then validated for plant growth promotion abilities under salinity stress conditions. This collection of microbes will be used in future experiments to help understand the beneficial effects of these endophytes on plant growth and stress tolerance promotion and for improving agricultural crop production in arid and hot regions.

## Materials and methods

### Soil analysis

30 mg of soil (triplicates) was used for soil analysis by drying thoroughly followed by nitric acid (1 M) digestion. Element measurement was performed using Inductively Coupled Plasma Optical Emission Spectrometry (Varian 720-ES ICP OES, Australia). The instrument conditions were: power (KW) 1.2, plasma flow (L/min) 1.65, auxiliary flow 1.5, nebulizer flow (L/min) 0.7, sample uptake delay (L/sec) 70, pump rate (rpm) 15 and rinse time (sec) 35.

### Study site description

Samples were collected from two sampling sites in Saudi Arabia; Jizan (16.8776N 42.6162E; 16.9412N, 42.6115E; 16.9405N, 42.6119E) and Al Wahbah (22.9017N, 41.1465E; 22.9084N, 41.1382E; 22.9070N, 41.1413E) which are approximately 650 kilometers apart (Panel A in S1 Fig). The Jizan province is located in the southern Red Sea coast and Al Wahbah Crater is located in a remote area in western Saudi Arabia as part of The Harrat extinct volcanic chain. The selected locations in the coastal habitats (Jizan) are characteristic of high humidity, reasonable rainfall and long summer days, while the inland habitats (Al Wahbah) are characteristic of low humidity, high evaporation rates and limited rainfall. The criteria of plant species collected was based on the plants being native/indigenous species to the region and adapted to its climate, perennials that persist for many growing seasons and woody shrubs and sub-shrubs with multiple stems arising at or near the base for easy access to the whole root system. In this study, no permissions were required to collect the samples as no such regulations apply in the Kingdom of Saudi Arabia and the field studies did not involve any endangered or protected species.

### Sample collection and bacterial isolation

The annual halophytes *Tribulus terrestris* and *Zygophyllum simpl*ex (both Zygophyllaceae) were collected from both locations. The C4 salt-tolerant perennial tussock-grass *Panicum turgidum* (Poaceae) and the prostrate, annual plant *Euphorbia granulata* (Euphorbiaceae) were only found in Jizan and, thus, collected from there. For each biological replicate, 3–4 root systems and their surrounding rhizosphere were collected in RNAlater solution (Sigma-Aldrich, Germany) and kept at ambient temperature. Plant roots stored in RNAlater were vortexed for 3 min to dislodge attached soil particles, and the detached soil was used for the analysis of the rhizosphere compartment. For the endosphere compartments, plant root were then washed for 10 sec in 70% ethanol then 20 sec in 2% sodium hypochlorite to remove attached microbes from root surface and, subsequently, washed twice with sterilized ddH_2_O [31], cut into small sections (0.5 mm in length), ground and used for both culture-dependent bacterial isolation and DNA extraction of endophytic bacterial communities.

### DNA extraction

For bacterial community profiling, DNA was extracted from plant root materials (endosphere), dislodged soil material (rhizosphere) and a soil sample collected away from vegetation to serve as a reference control for barren soil. An optimized mass of 2 g barren soil and rhizosphere soil (wet weight after removal of RNAlater) resulted in the best DNA quality and concentration. DNA extraction was performed using the PowerSoil DNA Isolation Kit (MO BIO Laboratories, Carlsbad, CA, USA) and the samples were subjected to 1 cycle of bead beating for 2 min at speed 2000 rpm using the PowerLyzer24 Homogenizer (MO BIO Laboratories, Carlsbad, CA, USA). For plant roots, 100 mg of root material (triplicate) was used for DNA extraction using the previously described bead beating program.

### 16S rRNA gene amplicon sequencing

Sequencing libraries of bacterial communities of soil, rhizosphere and root endosphere were prepared according to the Illumina 16S Metagenomic Sequencing Library Preparation guide. Briefly, the V3-V4 regions of the 16S bacterial rRNA gene were amplified using a two-step PCR protocol with V3-V4 primers (For: 5’-TCGTCGGCAGCGTCAGATGTGTATAAGAGACAG CCTACGGGNGGCWGCAG-3’; Rev: 5’-GTCTCGTGGGCTCGGAGATGTGTATAAGAGACAG GACTACHVGGGTATCTAATCC-3’, overhang adapter sequences are underlined) [32] for the first PCR step and Illumina Nextera XT Index kit (Illumina Inc., San Diego, CA, USA) for the second PCR step. The first PCR step (amplicon PCR) was performed in triplicate to amplify the 16S V3V4 region of the bacterial community using the above primer set with 2X KAPA HiFi HotStart ReadyMix (KAPA Biosystems, Woburn, MA, USA) in a total volume of 25 μl. The cycle conditions were as follows: initial cycle at 95°C for 3 min, 25 cycles of denaturation at 95°C for 30 sec, annealing at 55°C for 30 sec and 72°C for 30 sec with a final extension 72°C for 5 min. The triplicates were then pooled and used for the second PCR step (index PCR) which was carried out to attach the dual indexes (N7xx and S5xx) and Illumina sequencing adapters using 2X KAPA HiFi HotStart ReadyMix in a total volume of 50 μl. The PCR conditions were as follows: initial cycle at 95°C for 3 min, 8 cycles at 95°C for 30 sec, 55°C for 30 sec and 72°C for 30 sec with a final extension at 72°C for 5 min. Amplicons were cleaned using Agencourt AMPure XP (Beckman Coulter Inc., Brea, CA, USA) magnetic beads. Libraries were validated with Qubit dsDNA HS assay kit (Thermo Fisher Scientific, Wilmington, DE, USA) and Agilent 2100 bioanalyzer with the DNA 7500 kit (Agilent Technologies, Santa Clara, CA, USA) and quantified with qPCR using KAPA library quantification kit (KAPA Biosystems, Woburn, MA, USA). The library was normalized and sequenced at the KAUST Bioscience Core Labs on an Illumina MiSeq (Illumina Inc., San Diego, CA, USA) with 2 x 300 bp paired-end reads and V3 chemistry.

### Data processing and analysis

The software mothur (version 1.36.1) [33] was used for 16S rRNA sequence editing and analysis following the pipeline in Röthig, Roik [34]. Briefly, reads were demultiplexed and sequences were quality trimmed, followed by a pre-clustering step (2 bp difference) [35], removal of singletons and alignment against the SILVA database (release 119) [36]. Chimeric sequences were removed using UCHIME [37] and reads assigned to chloroplasts, mitochondria, archaea and eukaryotes were excluded. Sequences were classified against Greengenes database (release gg_13_8_99; bootstrap = 60) [38].

Phylogenetically classified sequences were used to create bacterial community composition stack column plots at the phylum and family level using the means of relative abundances from replicated samples (n = 2 or 3). For further analyses, sequences were subsampled to 955 sequences per sample and clustered into Operational Taxonomic Units (OTUs), using a 97% similarity cutoff. The most abundant sequence of each OTU was selected as representative OTU sequence. Alpha diversity measures (number of OTUs, Chao estimate of species richness and Simpson diversity and evenness) were calculated separately for all plant species and sample types at each location. Non-parametric Kruskal-Wallis tests were performed to test for differences in species richness, diversity and evenness between sample types (endophyte, rhizosphere, soil) and locations (Jizan, Al Wahbah).

Multivariate analyses of beta diversity based on Bray-Curtis distances were conducted using PRIMER-E v6 (PERMANOVA+) software package [39]. Permutational MANOVA (PERMANOVA) was used to test and non-Metric Multidimensional Scaling (nMDS) was used to visualize differences between ‘species and soil’ (5 levels: E, P, T, Z and S), ‘type of sample’ (3 levels: endosphere, rhizosphere, soil) and ‘site’ (2 levels: Jizan, Al Wahbah). All multivariate tests were performed on square root-transformed data of OTU counts with PERMANOVA, using partial sum of squares and 9,999 permutations under a reduced model. Analyses are based on OTU counts (for OTUs ≥ 10 reads).

### Media and culture conditions for retrieval of bacterial isolates from endosphere of Jizan desert plants

Surface sterilized roots were macerated with 0.8% saline solution, the liquid homogenate was diluted in 0.8% saline solution and 10^−4^ and 10^−5^ dilutions were used as inoculum for bacterial isolation. Four main culture media were used for the purification of bacterial isolates, namely Luria-Bertani (LB) agar (Sigma Aldrich, Germany), BD Difco R2A (R2A) agar (BD Diagnostics, Sparks, MD, USA) with and without 1.5 or 3% of added sodium chloride (NaCl) and Tryptone Soya Agar (TSA) agar (g/L: tryptone-15; soytone-5; NaCl-5; agar-15). 100 μl of diluted root extract was spread on different agar plate’s media. Inoculated plates were incubated at 28°C for 3–4 days and isolated colonies were purified by re-streaking until pure culture was achieved. Purified bacterial isolates were stored in 20% glycerol at -80°C. These isolates were then used for 16S classification, screening of biochemical traits and growth promotion of Arabidopsis.

### Identification and taxonomic assignment of culturable bacteria

The amplification of the 16S rRNA gene fragment was carried out using Taq DNA polymerase PCR Master Mix (Promega, Madison, WI, USA) with universal primer sets 27F and 1492R (27F: 5’-AGAGTTTGATCCTGGCTCAG-3’ and 1492R: 5’-TACGGYTACCTTGTTACGACTT-3’). The PCR amplification of 16S rRNA genes was carried out using the following PCR conditions: an initial denaturation at 95°C for 1 min, followed by 30 cycles with steps of 95°C for 30 sec, 55°C for 45 sec and 72°C for 90 sec and a final extension step of 5 min at 72°C. Amplification was confirmed by analyzing PCR products on a 1% agarose gel. PCR products were purified from incorporated primers and extra dNTPs using ExoSAP-IT (Affymetrix, Santa Clara, CA, USA) and sequenced using ABI 3730xl DNA Analyzer (Applied Biosystems, Foster City, CA, USA). The 16S rRNA gene sequences of the bacterial isolates were compared to known sequences listed in NCBI’s GenBank using BLAST [40]. Proposed taxonomic assignment of culturable bacteria were based on BLAST annotation using sequence identity and query cover as main criteria. The sequences were also annotated with Greengenes to allow comparison with MiSeq data.

### Bioassays for plant growth promoting traits and tolerance to abiotic stresses

Calcium phosphate solubilization ability of bacteria was determined based on formation of clear halo on Pikovskaya’s (PVK) (g/L: yeast extract-0.5; dextrose-10; calcium phosphate-5; ammonium sulphate-0.5; potassium chloride-0.2; magnesium sulphate-0.1; manganese sulphate-0.0001; ferrous sulphate-0.0001; agar-15) agar plates (M520, Himedia, France) [41]. Siderophore production were determined by formation of orange halo on CAS agar plates as described by Louden, Haarmann [42], with slight modification. The casamino acids were extracted with 3% (w/v) 8-quinolinol hemisulphate salt instead of 8-hydroxyquinoline, and the phases were separated overnight at 4°C.Zinc oxide (ZnO) or carbonate (ZnCO_3_) solubilization was evaluated following Bapiri et al. [43], by clearing assay using a modified PVK (g/L: yeast extract-0.2; glucose-10; ammonium sulphate-1.0; potassium chloride-0.2; dipotassium hydrogen phosphate-0.2; magnesium sulphate-0.1; agar-15) agar plates modified with 0.1% (w/v) zinc oxide (ZnO) or carbonate (ZnCO_3_). All clearing assays were performed in triplicates by inoculating 30 μl of overnight bacterial culture into cavities of ~0.5 cm in diameter. Assay plates were then incubated at 28°C for 3–5 days. IAA production was qualitatively determined according to Bric et al. [44], however, either liquid LB or King’s B (g/L: Difco peptone-20; dipotassium hydrogen phosphate-1.15; MgSO_4_.7H_2_O-1.5; and glycerol-1.5% w/v) media modified with 2.5 mM L-Tryptophan (Sigma Aldrich, Germany) were used instead of agar plates. The media was inoculated with 1 μL of overnight culture and incubated at 28°C and 190 rpm for 2 days in 96-well plates. Formation of pinkish to red color indicated positive for IAA production. Tolerance to abiotic stresses was assessed by growing the isolate in liquid culture for 2 days at 28°C and 190 rpm. For drought and salinity stress, media was supplemented with 20% Polyethylene-glycol (PEG) 8,000 and 5% NaCl, respectively.

### Plant assays

*Arabidopsis thaliana* Col-0 (wild-type) seeds were surface sterilized by shaking for 10 min in 70% ethanol + 0.05% sodium dodecyl sulfate (SDS), then washed twice with 99% ethanol and once with sterilized H_2_O. The seeds were then sown on square Petri dishes (12x12 cm) containing half-strength Murashige and Skoog Basal Salt Mixture pH 5.8, 0.9% agar (½MS) [45] (M5524, Sigma Aldrich, Germany) without sucrose. The plates were stored in the dark for 2 days at 4°C for seed stratification and then incubated vertically (~75° angle to the horizontal) in growth chambers (Percival Scientific Inc., USA) at 22°C with a photoperiod of 16/8 h (light/dark) for germination. 5-day old seedlings (~1–1.5 cm in root lengths) were then gently transferred to fresh ½MS agar plates supplemented with 100 mM NaCl as a salinity stress (5 seedlings/plate). A “lawn” of bacterial isolates were spread on LB agar plates and incubated at 28°C 24 hours prior to transfer of seedlings. From these plates, square-shaped (3x3 mm) plugs were cut out and laid beside the root system of each seedling without any physical damage (bacteria-free LB agar plugs were used as a mock control) (Panel D in S1 Fig). For assessment of the effect of inoculation with cultured bacteria, images of representative plants were taken 16 days after transfer (DAT) and compared to mock (bacteria free LB control).

### Statistical analysis

The data from the plant screening assay were subjected to non-parametric one-way ANOVA, or Kruskal-Wallis H test [46]. Data were expressed as the mean ± standard error of the mean (SEM). The differences among the various treatment means were compared and the values with a *p* value of ≤ 0.05 were considered statistically significant (as indicated by asterisks). Statistical analysis was done using DEVELVE statistical software (https://www.develve.net/). The statistical analysis for soil nutrient analysis was done using Student’s t-test (*p* ≤ 0.05).

### Accession numbers

The 16S rRNA gene sequences of the bacterial isolates in this study have been deposited in the GenBank database and are accessible under accession numbers (KY194215—KY194330). MiSeq data determined in this study are available at NCBI under the BioProject ID PRJNA431874.

## Results

### Sample collection and soil physicochemical properties

The two sampling sites contained soil with significantly different properties (Table 1). The site at Jizan contained coarse-sandy soil with neutral pH of 7.19, while the site at Al Wahbah was sandy with a slightly alkaline pH of 8.50. Soil from Al Wahbah was lower in phosphate and potassium but richer in calcium and magnesium than the soil from Jizan. In terms of micronutrients, both soils contained similar amounts of boron, copper, iron and zinc. However, manganese was relatively higher in Al Wahbah than Jizan. Other elements were mostly equivalent in both soils, except for sodium, nickel and strontium, which were higher in Al Wahbah.

**Table 1:** Site description and physicochemical properties and elemental composition of soil from two sampling sites (Jizan, Al Wahbah) in Saudi Arabia.

	Site A	Site B
Location	Jizan	Al Wahbah
**Latitude; Longitude**	16.9405N, 42.6119E	22.9070N, 41.1413E
**Total precipitation per year (mm)**	301	91
**Maximum temperature (°C)**	38	44
**Average temperature (°C)**	30	33
**Minimum temperature (°C)**	26	12
**Color and texture**	Brown with black particles; coarse-sandy	Dark brown; Sandy
**pH**	7.19	8.50
**Moisture content (%)**	21.5	17.8
**Macronutrients**		
**P (g Kg^-1^)**	0.72 ± 0.05*	0.55 ± 0.02
**K (g Kg^-1^)**	2.92 ± 0.15*	1.22 ± 0.26
**Ca (g Kg^-1^)**	3.38 ± 0.3*	11.13 ± 0.59
**Mg (g Kg^-1^)**	6.35 ± 0.29*	10.24 ± 0.42
**Micronutrients**		
**B (mg Kg^-1^)**	7.39 ± 0.57	9.89 ± 1.17
**Cu (mg Kg^-1^)**	13.67 ± 0.17*	19.83 ± 1.19
**Fe (mg Kg^-1^)**	1664.68 ± 26.5	1742.11 ± 77.52
**Mn (mg Kg^-1^)**	226.19 ± 15.66*	320.64 ± 11.59
**Zn (mg Kg^-1^)**	31.65 ± 7.21	37.54 ± 1.12
**Trace Elements**		
**Al (mg Kg^-1^)**	8068.28 ± 162.2*	7645.95 ± 99.46
**Ba (mg Kg^-1^)**	75.59 ± 9.83	59.07 ± 2.04
**Ce (mg Kg^-1^)**	13.65 ±1.36	12.00 ± 0.98
**Cr (mg Kg^-1^)**	32.52 ± 6.41	23.13 ± 2.58
**Na (mg Kg^-1^)**	195.97 ± 6.20*	450.64 ± 32.25
**Ni (mg Kg^-1^)**	22.63 ± 6.54*	49.27 ± 3.71
**Pb (mg Kg^-1^)**	18.79 ± 4.97	18.73 ± 0.51
**Sr (mg Kg^-1^)**	26.37 ± 5.95*	76.31 ± 1.93
**Ti (mg Kg^-1^)**	848.93 ± 63.61	786.17 ± 11.74
**V (mg Kg^-1^)**	38.24 ± 6.40	37.25 ± 2.11

Data presented are mean values (± standard deviation) of three independent replicates, and asterisks indicate statistically significant differences (Student’s t-test, *p* <0.05).

### Amplicon sequencing of bacterial communities

Bacteria from different fractions (soil, rhizosphere, and endosphere) from four different desert plants were analyzed by sequencing the V3-V4 region of the 16S rRNA gene. MiSeq sequencing yielded 9,276,890 sequences with a mean length of 445 bp. After quality filtering, exclusion of chimeras and amplified plant mitochondria, 565,007 sequences were annotated to bacteria. For further analysis, sequences were clustered into Operational Taxonomic Units (OTUs), using a 97% similarity cut off, resulting in 2,704 OTUs.

**Fig 1:**
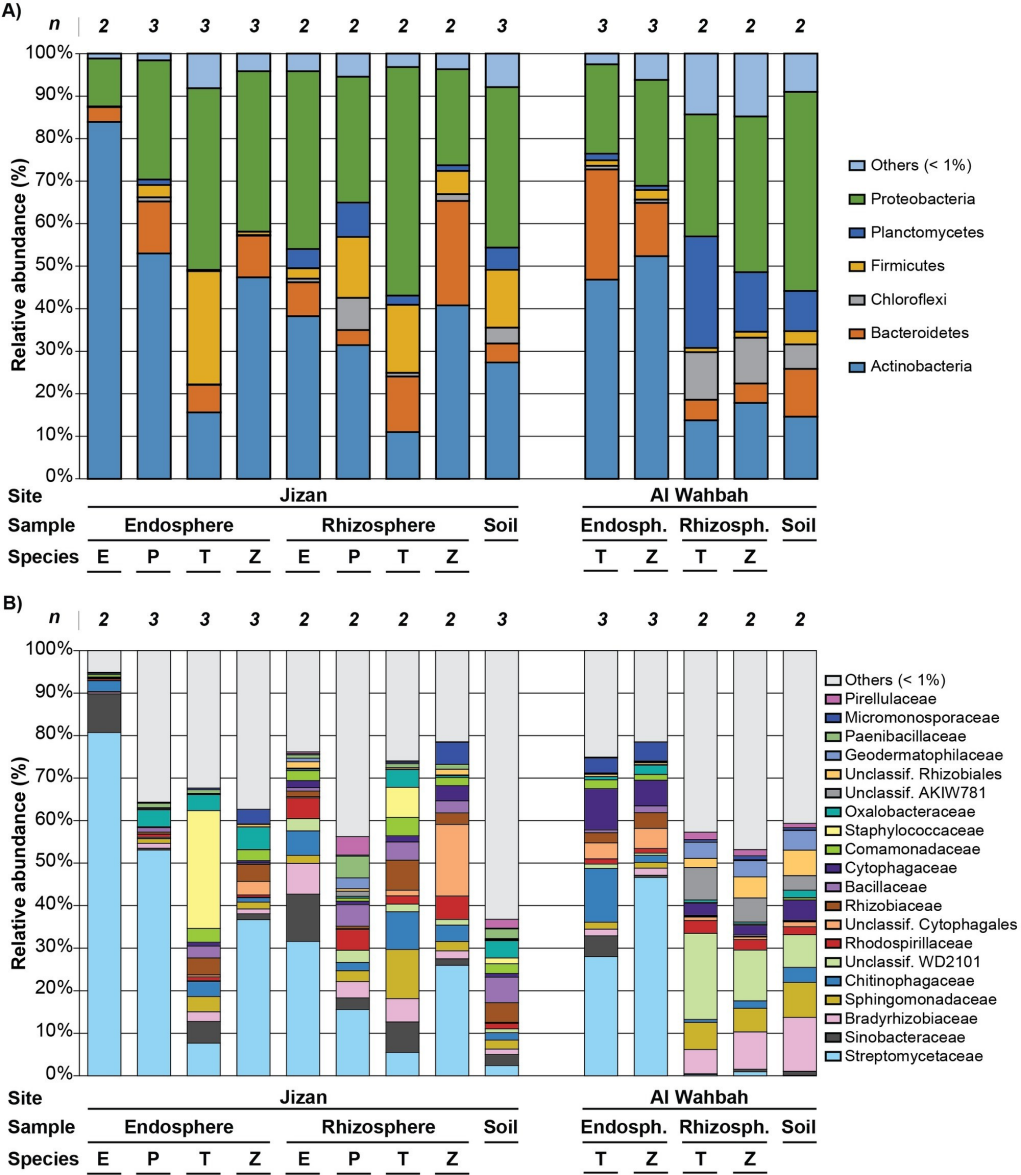
Taxonomic composition of the soil, rhizosphere and root endosphere of pioneer desert plants in Jizan and Al Wahbah. Relative abundance of bacterial phyla **(A)** and bacterial families **(B)** associated with the soil, rhizosphere and root endosphere of four plant species at two different locations of the Saudi Arabian desert (Jizan and Al Wahbah), based on the V3-V4 region of the 16S rRNA region. Number of biological replicates indicated above stacked columns **(*n*)**, taxa present at greater than 1% of the average community are shown. E—*E*. *granulata*; P—*P*. *turgidum*; T—*T*. *terrestris*; Z—*Z*. *simplex*.

Bacterial communities were dominated by seven phyla with Actinobacteria and Proteobacteria being most abundant over all sample types (Fig 1A). The phylum Firmicutes was present at higher proportions in samples from Jizan compared to Al Wahbah, while Planctomycetes were primarily associated with soil and rhizosphere samples from Al Wahbah. The abundance of Actinobacteria was gradually increased from soil to rhizosphere to endosphere in Jizan samples (except for *T*. *terrestris*). *T*. *terrestris* from Jizan was, however, enriched in Firmicutes in the endosphere. The rhizospheres of Al Wahbah plants were enriched in Planctomycetes, while the endosphere was enriched with Actinobacteria and Bacteroidetes.

Twenty bacterial families were associated with desert soil and plant roots at > 1% abundance (Fig 1B). Root endosphere samples from both sites were dominated by the family Streptomycetaceae (28–81%), except in *T*. *terrestris* from Jizan where Staphylococcaceae was dominant (27.7%). All other bacterial families were relatively evenly distributed across samples.

### Bacterial diversity of plants and soil

Alpha diversity measures (number of OTUs, Chao estimate of species richness, Simpson diversity and evenness) were calculated separately for each species, sample type and location (Table 2). Soil samples had overall highest species richness among all samples, followed by rhizosphere samples, which were both significantly higher than endosphere samples (Kruskal-Wallis H = 19.92, *p* < 0.001). Endosphere samples also had significantly lower species diversity (Kruskal-Wallis H = 16.99, *p* < 0.001) and evenness (Kruskal-Wallis H = 11.0, *p* < 0.005) compared to soil and rhizosphere samples. Generally, samples from Al Wahbah had higher bacterial species richness (Kruskal-Wallis H = 7.35, *p* < 0.01) than samples from Jizan, while species diversity and evenness were not significantly different between sites (Kruskal-Wallis, *p* > 0.05).

**Table 2:** Summary of average alpha diversity measures of bacterial communities associated with plant root endosphere (Endo), rhizosphere (Rhizo) and soil samples at each site (Jizan, Al Wahbah).

Site	Species	Sample (*n*)	# of OTUs	Chao1 Estimator	Inverse Simpson’s Metric	Simpson’s Evenness
**Jizan**	E	Endo (2)	45.0 ± 2.8	96.1 ± 14.3	1.65 ± 0.22	0.037 ± 0.007
		Rhizo (2)	215.0 ± 18.4	444.0 ± 58.5	10.21 ± 2.02	0.047 ± 0.005
	P	Endo (3)	86.0 ± 5.7	148.6 ± 3.7	4.37 ± 4.02	0.053 ± 0.050
		Rhizo (2)	236.7 ± 84.6	407.6 ± 216.3	30.65 ± 18.31	0.120 ± 0.043
	T	Endo (3)	119.3 ± 24.2	151.2 ± 14.9	14.97 ± 11.67	0.117 ± 0.087
		Rhizo (2)	188.5 ± 4.9	387.2 ± 23.6	30.11 ± 0.16	0.160 ± 0.003
	Z	Endo (3)	90.7 ± 33.3	128.9 ± 53.3	9.33 ± 6.75	0.107 ± 0.065
		Rhizo (2)	163.0 ± 2.8	296.7 ± 3.7	13.13 ± 0.51	0.081 ± 0.002
	S	Soil (3)	265.8 ± 73.7	498.5 ± 102.7	74.01 ± 44.37	0.251 ± 0.134
**Al Wahbah**	T	Endo (3)	168.0 ± 20.1	384.5 ± 81.1	13.23 ± 8.82	0.078 ± 0.051
		Rhizo (2)	338.5 ± 60.1	752.8 ± 156.4	96.90 ± 35.4	0.281 ± 0.055
	Z	Endo (3)	156.3 ± 20.6	277.7 ± 3.3	6.87 ± 3.79	0.042 ± 0.018
		Rhizo (2)	436.5 ± 13.4	1165.3 ± 11.3	107.05 ± 10.27	0.245 ± 0.016
	S	Soil (2)	375.0 ± 33.9	871.1 ± 87.6	59.62 ± 9.74	0.158 ± 0.012

E—*E*. *granulata*; P—*P*. *turgidum*; T—*T*. *terrestris*; Z—*Z*. *simplex*; S—Soil; *n*—number of samples. Data presented are mean values (± standard deviation).

### Differences between soil and plant compartments and collection sites

**Fig 2:**
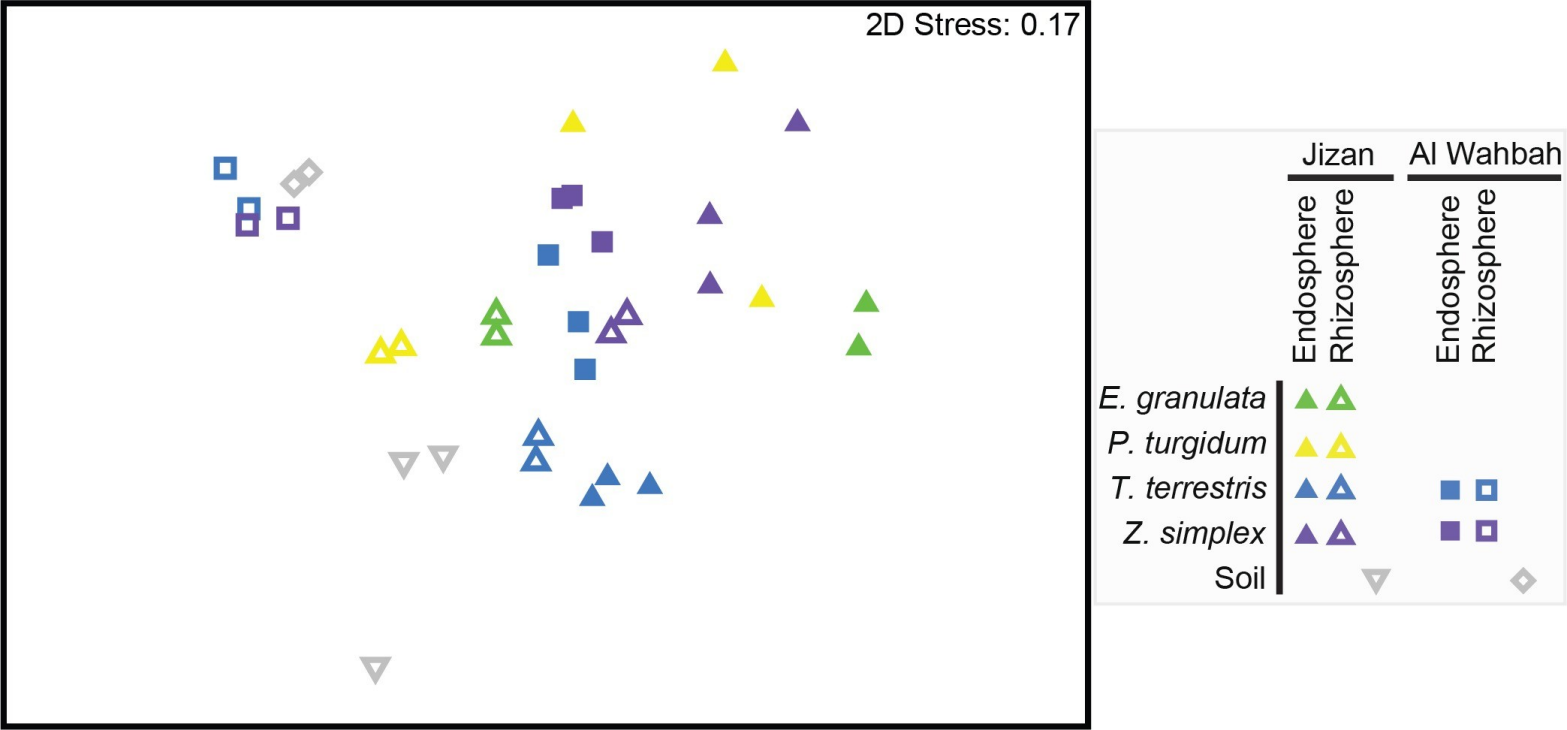
Non-metric multidimensional scaling (nMDS) of bacterial communities from the soil, rhizosphere and root endosphere of four pioneer desert plants in Jizan and Al Wahbah. The plot is based on Bray Curtis distances of square root transformed abundance data of bacterial OTUs (for OTUs > 9 reads). The stress value denotes the goodness of fit. Plant species and soil samples are indicated by colors: green (*E*. *granulata*), yellow (*P*. *turgidum*), blue (*T*. *terrestris*), purple (*Z*. *simplex*), grey (soil); sample locations are indicated by shapes: triangles (Jizan), squares (Al Wahbah); sample types are indicated as: filled symbols (endosphere), hollow symbols (rhizosphere).

Based on results from NMDS and PERMNOVA, bacterial communities were significantly different between sites at Jizan and Al Wahbah (PERMANOVA, F = 4.1, p(MC) < 0.05). Further, bacterial communities were distinct between sample types (i.e., rhizosphere, endosphere) (PERMANOVA, F = 4.0, p(MC) < 0.001), with rhizosphere samples clustering with soil samples collected at the same site (Fig 2). Endosphere samples from the same plant species clustered together regardless of site and we found significant differences between rhizosphere and endosphere bacterial communities for *E*. *granulata* (pairwise t = 2.7, *p* < 0.05), *T*. *terrestris* (pairwise t = 2.3, *p* < 0.01) and *Z*. *simplex* (pairwise t = 2.7, *p* < 0.01). In *P*. *turgidum*, rhizosphere and endosphere communities were not significantly different (*p* > 0.05), but this could possibly be associated with the low sample replication for this species (n = 2) as one set of samples did not pass quality requirements.

### Taxonomic composition of culturable bacterial endophytes from Jizan desert plants

**Fig 3:**
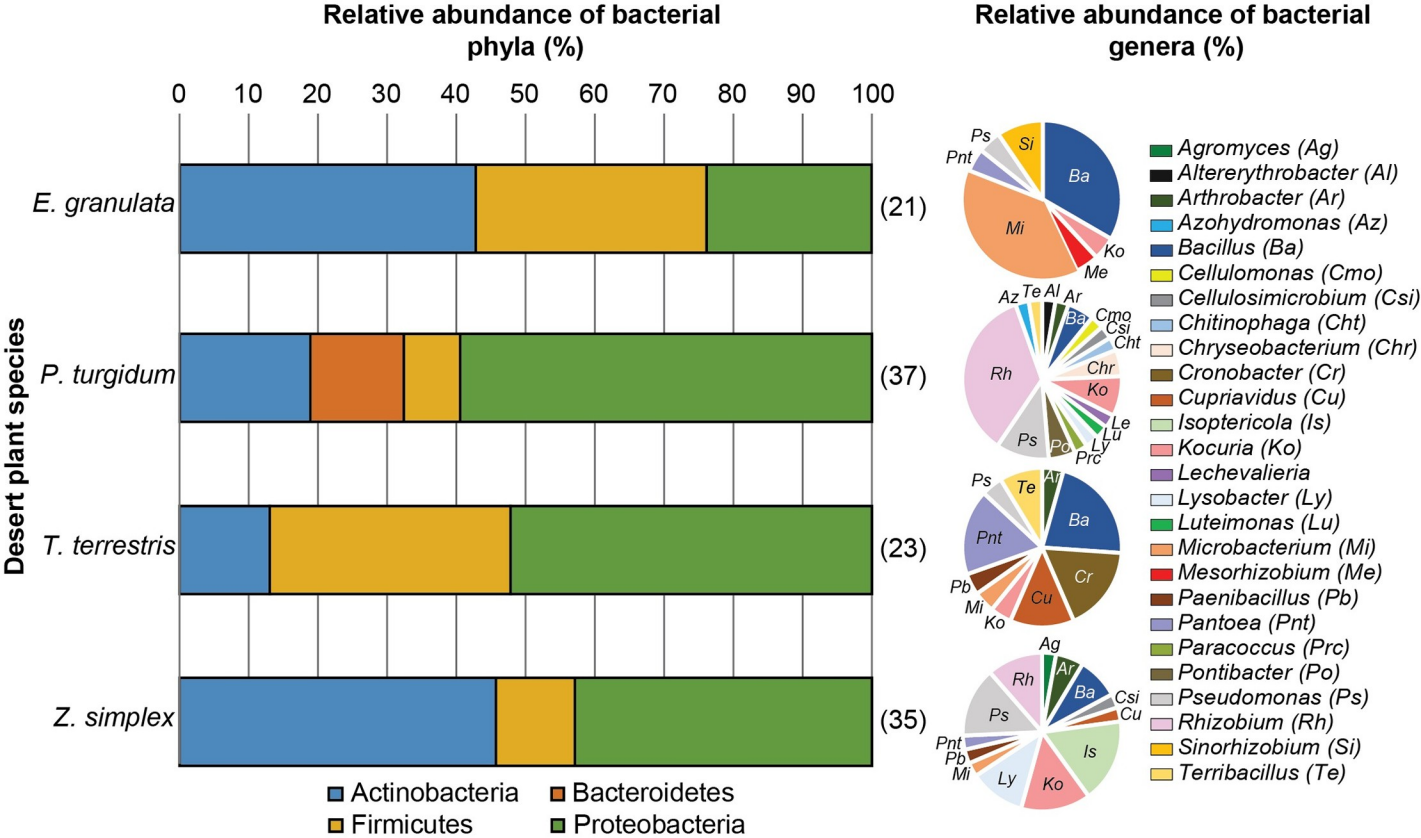
Taxonomic composition of culturable root endosphere bacteria (endophytes) from Jizan desert plants. Relative abundance of the bacterial phyla (bar chart) and genera (pie chart) as a percentage of the total bacteria isolated from each plant species’ root endosphere (presented after each bar in parentheses), based on the full-length 16S rRNA sequences.

Bacteria were isolated from the root endosphere (endophytes) of the four desert plants from Jizan. Isolation of 116 bacteria from the four plant species (Euphorbia, 21; Panicum, 37; Tribulus, 23; and Zygophyllum, 35 isolates) was achieved by using a plate dilution method on different synthetic growth media; 49% of bacterial isolates were obtained from R2A with 1.5–3% NaCl, 28% from TSA and 22% from LB agar plates. The bacterial isolates displayed a variety of morphological features in terms of color, size and shape. The phylogenetic classification of the culturable bacteria from the Jizan collection (Fig 3) revealed a variation in phyla that was largely overlapping with the 16S amplicon sequencing data (Fig 1).

Proteobacteria were the most cultivatable phylum with 54 bacterial isolates, while Bacteroidetes were the least cultivatable with only 5 isolates. Actinobacteria and Firmicutes contained 35 and 22 isolates, respectively. Proteobacteria were highly dominant in *P*. *turgidum* (59%) and *T*. *terrestris* (52%), while Actinobacteria were dominant in *Z*. *simplex* (46%) and *E*. *granulata* (43%) (Fig 3, bar chart). Bacteroidetes were only present in the isolates from *P*. *turgidum*.

The isolates belonged to different genera with the most abundant genera being *Rhizobium*, *Bacillus*, *Pseudomonas*, *Kocuria* and *Microbacterium*. The highest number of bacterial genera (Fig 3, pie chart) was found in the root system of *P*. *turgidum* (17), followed by *Z*. *simplex* (13), *T*. *terrestris* (10) and *E*. *granulata* (7). Some bacterial genera were present in all plant species, such as *Bacillus*, *Kocuria* and *Pseudomonas*. Whereas some were found only in one particular plant species, such as *Cronobacter* in *T*. *terrestris*, *Agromyces* in *Z*. *simplex*, *Mesorhizobium and Sinorhizobium* in *E*. *granulata* and Bacteroidetes and other genera (e.g. *Paracoccus*, *Azohydromonas*) found specifically in *P*. *turgidum*.

### Qualitative assessment of PGP traits and survival under abiotic stresses

The bacterial isolates were tested for survival in salinity and drought stress conditions and a number of biochemical properties such as the solubilization of calcium phosphate, ZnO and ZnCO_3_, or the production of siderophores and IAA.

**Fig 4:**
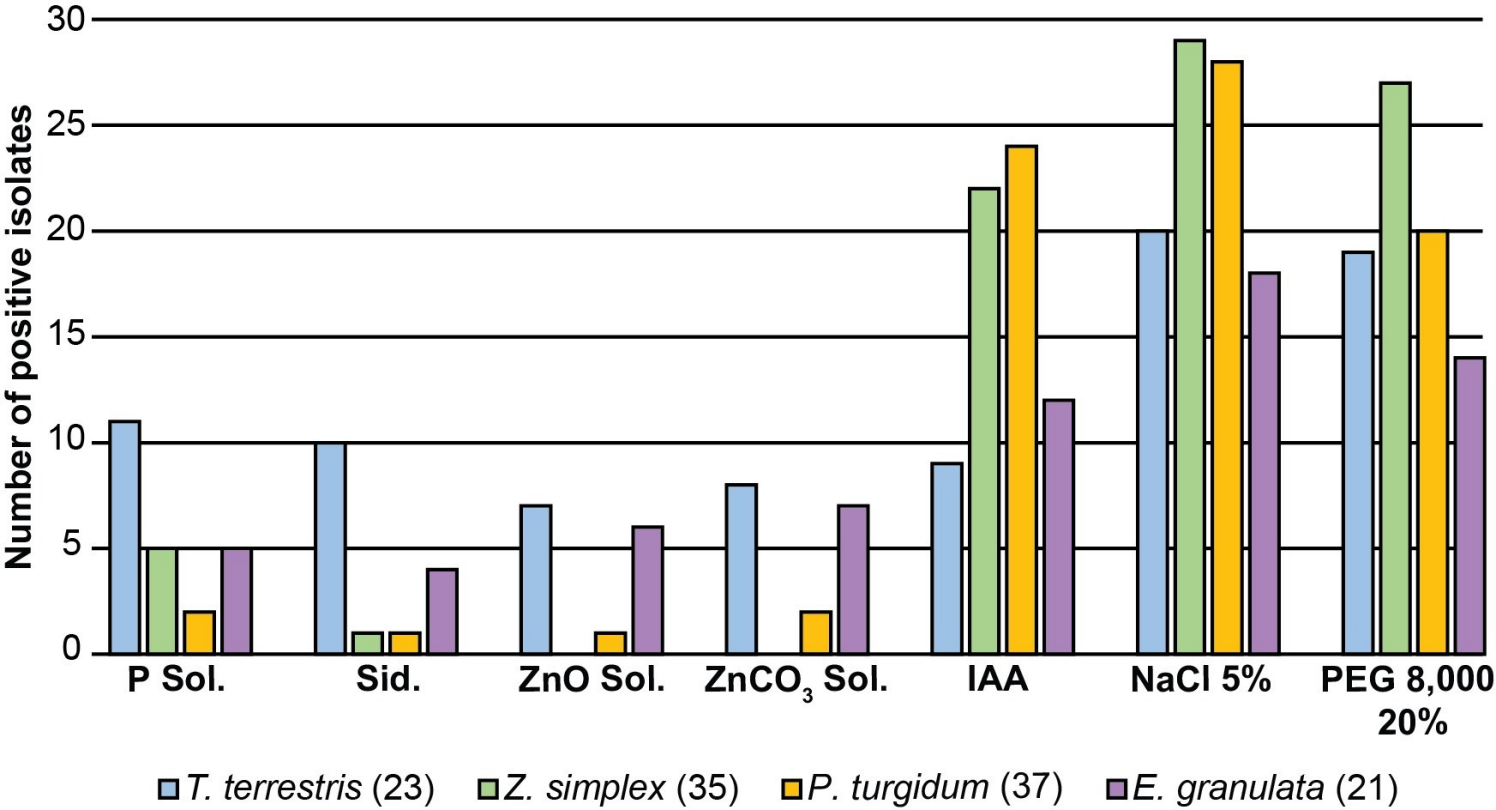
Culturable bacterial endophytes from Jizan desert plants possess PGP traits and ability to survive in abiotic stresses. Abundance of bacteria with PGP traits and ability to survive in abiotic stresses in each desert plant species on qualitative biochemical assays. P Sol.—calcium phosphate solubilization; Sid.—siderophore production; ZnO, ZnCO_3_ Sol.—zinc oxide/carbonate solubilization; IAA—indole-acetic acid production; NaCl 5%—growth on 5% NaCl; PEG 8,000 20%—growth on 20% PEG 8,000. Total bacteria isolated from each plant species’ root endosphere is presented in parentheses.

As shown in Fig 4 (for details see S2 Fig), out of the 116 bacterial isolates, most of the isolates survived on LB plates supplemented with 5% NaCl (95 isolates) and 20% PEG 8,000 (80 isolates). In terms of nutrient acquisition, 23 strains were able to solubilize calcium phosphate, 16 were able to produce siderophores and 14 and 17 possessed ZnO and ZnCO_3_ solubilization abilities, respectively (S2 Fig). *T*. *terrestris* showed the highest abundance of bacterial isolates with PGP traits related to nutrient acquisition (calcium phosphate, 11 isolates; ZnO solubilization, 7; ZnCO_3_ solubilization, 8; and siderophore production, 10), followed by *E*. *granulata* (5, 6, 7 and 4 isolates) (Fig 4A). In contrast, *P*. *turgidum* and *Z*. *simplex* retrieved much fewer isolates with nutrient acquisition traits. However, these plants had the highest abundance of IAA producing bacteria (24 and 22 isolates, respectively).

### Promotion of salinity stress tolerance in Arabidopsis

Endophytic bacterial isolates from Jizan plants were screened for their ability to enhance the salinity stress tolerance of the model plant *A*. *thaliana* under 100 mM NaCl. The selection of salinity stress tolerance (SSTP) was based on the criteria whether the isolates could positively affect plant shoot biomass when compared to non-inoculated mock (bacteria free LB control) plants. The qualitative screen revealed that 11 isolates exhibited SSTP abilities (S3 Fig), while the rest had a negative or no significant impact on the shoot biomass when compared to mock plants.

The bacterial strains that exhibited positive growth on shoot biomass of *A*. *thaliana* plants under salinity stress conditions belonged to the following families: Bacillaceae (JZ34), Paenibacillaceae (JZ16), Oxalobacteraceae (JZ4), Micrococcaceae (JZ12), Microbacteriaceae (JZ31 and JZ37), Promicromonosporaceae (JZ7 and JZ28), and Enterobacteriaceae (JZ2, JZ29, and JZ38). These families were present at less than 1% of the total endosphere bacterial communities, except for Paenibacillaceae (1.05% in *P*. *turgidum*), Bacillaceae (1.04% in *P*. *turgidum* and 2.78% in *T*. *terrestris*), and Oxalobacteraceae (3.77%, 3.99%, and 5.31% in *T*. *terrestris*, *P*. *turgidum*, and *Z*. *simplex*, respectively). Isolates JZ29 and JZ38 were found to possess all PGP traits and abilities in addition to promotion of salinity stress tolerance in *A*. *thaliana*.

**Fig 5:**
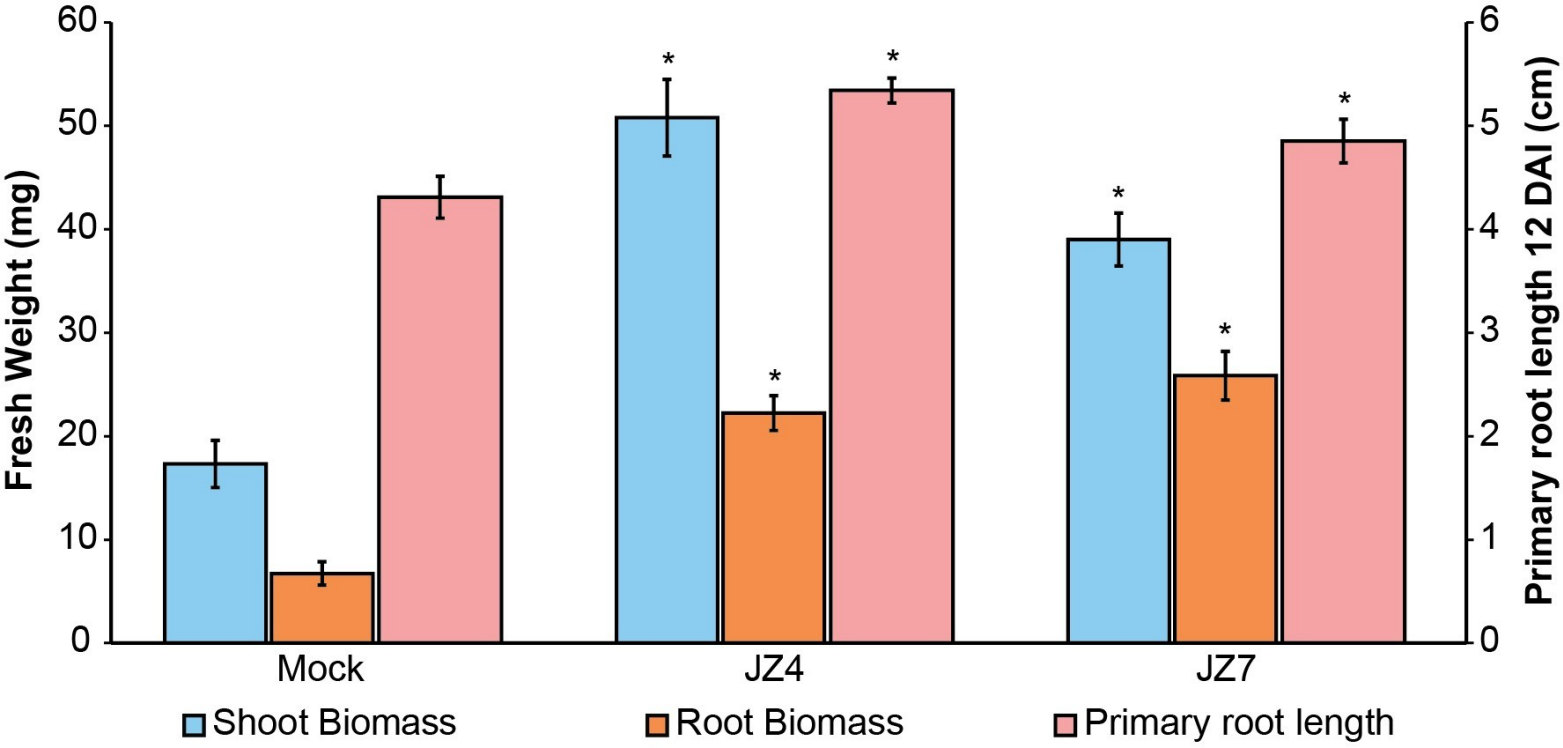
Effect of bacterial inoculation on *A*. *thaliana* growth under salinity stress. Quantitative measurement of fresh weight of *A*. *thaliana* plant shoots and roots collected 16 DAI (days after inoculation) and the effect of bacterial inoculation on the primary root length measured 12 DAI. Data are means of 4 biological replicates of at least 8 plants per treatment. Error bars represent standard error of the mean (SEM). Asterisks indicate significant differences between Control (100 mM NaCl) and JZ-inoculated/non-stressed (0 mM NaCl) plants (Kruskal–Wallis test, *p* < 0.05).

Next, quantitative measurements were performed on shoot and root biomass (fresh weight) and primary root length of plants inoculated with isolates JZ4 and JZ7 (Fig 5). The taxonomic classification of JZ4 and JZ7 were *Cupriavidus gilardii* and *Isoptericola variabilis*, respectively. Under salinity stress, isolates JZ4 and JZ7 increased the shoot fresh weight by 193% and 125%, respectively. The isolates also increased root system biomass (JZ4, 230%; JZ7, 283.7%) and led to longer primary roots (JZ4, 23.9%; JZ7, 12.6%), compared to mock control plants.

## Discussion

The plant root endosphere is usually dominated by a small number of bacterial lineages, with Actinobacteria and Proteobacteria being the dominant phyla when compared to soil and rhizosphere bacterial communities. This observation was made for a number of crop plants, such as maize, barley, rice and grapevine [47–50], and also in the model plant *Arabidopsis thaliana* [51] and a number of desert plants such as *Agave*, *Atriplex*, *Tribulus* and *Zygophyllum* [52–54]. Our analysis of the physicochemical properties and bacterial communities of the soils from Jizan and Al Wahbah in the Saudi Arabian desert revealed significant differences in the quantity of macro- and micronutrients that was reflected in the bacterial diversity at these sites. By examining the rhizosphere and endosphere of the collected pioneer plants at the two sites, a dominance of Actinobacteria and Proteobacteria was observed in all samples, with Actinobacteria gradually increasing in abundance from soil to rhizosphere and finally to endosphere. Based on the alpha and beta diversity results, Al Wahbah samples were more diverse and species rich than Jizan samples. However, the general pattern of lower richness, diversity and uniformity in endosphere samples was conserved.

Plants are known to recruit microbial communities by releasing root exudates to the rhizosphere, thereby providing a major source of carbon and nutrients. The microbial communities recruited, which form the root microbiome, are dependent on a number of factors, such as the genotype of the host plant [47, 55, 56]. However, the primary determinant of the root-associated bacterial community was found to be the soil type, while the genotype played a secondary factor in the root microbiome composition [51, 57]. Our results agree with these findings and show that soil and rhizosphere microbiomes are closely correlated, whereas endosphere samples from the same plant genotype cluster together regardless of the geographical location. These results indicate that the host plant phylogeny aligns to a large degree with the composition of the endosphere bacterial community, and only to a lesser degree with that of the rhizosphere, which is largely determined by the soil.

Under desert conditions, pioneer desert plants develop intimate and often highly coevolved interactions with the soil microbes to achieve an adequate strategy for survival [18]. Based on our observations and due to its lower bacterial diversity, it was hypothesized that the endosphere compartment would contain bacteria with the ability to colonize, interact and support plants in their ability to tolerate abiotic stresses. Therefore, bacteria were isolated from the root endosphere of desert plants and investigated further.

The bacterial isolates were tested for several PGP traits and abiotic stress tolerance abilities. Bacteria belonging to different phyla displayed various PGP traits and abilities to tolerate salt and drought stress. At the phylum level, Proteobacteria (especially those of the γ-class) contained the highest proportion of bacteria with PGP abilities. Among them, the genus *Cronobacter* and *Pantoea* showed the highest number and proportion of PGP traits in our bacterial collection. Indeed, different species of the *Pantoea* and *Cronobacter* genera isolated from different host plants were already shown to possess PGP abilities [58–62]. Thus, species belonging to these genera may be highly adapted to salinity and/or drought stress.

Some bacterial isolates from different plant species were identical at the species level, with 99% sequence identity based on 16S analysis, but exhibited different PGP abilities. For example, two *P*. *stewartii* strains JZ2 and JZ29 were isolated from different plants and possessed different PGP traits and abilities. Our results suggest that some bacterial isolates may have undergone host-specific adaptations and gained strain-specific traits.

Beyond the natural plant host, the isolated bacteria were tested for their potential effect on the growth of the model plant *A*. *thaliana* under abiotic stress conditions. The SSTP screening assay used in this study was based for the inoculation of plants with the corresponding isolates. Our assay offers the ability to screen for both direct and indirect interactions and modes by which bacterial isolates induce salinity stress tolerance in *A*. *thaliana*. In direct interactions, direct contact/colonization is required whereby indirect/contactless interactions can be mediated by small molecules, e.g. by emission of volatile compounds. It has been demonstrated that bacteria are able to induce abiotic stress tolerance via the emission of volatile organic compounds (VOCs) [63–65]. The plant assays identified 11 bacterial strains that exhibited a positive effect on the shoot biomass of the model plant *A*. *thaliana* under salinity stress conditions. These strains were not taxonomically related and belonged to different phyla, families and genera. For example, three salinity stress tolerance promoting bacteria (JZ29, JZ31 and JZ34) isolated from *E*. *granulata* were representatives of the Actinobacteria, Firmicutes and Proteobacteria phylum. Similarly, the four bacteria JZ2, JZ4, JZ37 and JZ38, isolated from *T*. *terrestris*, were representatives from the Enterobacteriaceae, Burkholderiaceae and Microbacteriaceae family. This indicates that the ability of the bacteria to promote salinity stress tolerance is not an exclusive property of one specific phyla, family or genera. Instead, a number of different bacteria seem to possess plant growth promoting mechanisms and can engage in symbiotic relationships with the host plants under extreme conditions. When comparing the abundance of these strains with the total bacterial community in the root endosphere, we found that the SSTP bacteria are rare symbionts that may play very crucial, but specific, roles in plant-microbe interactions.

In order to obtain a deeper understanding of the salinity stress tolerance mechanisms induced by PGPB, the 11 strains that exhibited positive effects on the shoot fresh weight are currently being further characterized at both the genomic and transcriptome levels. The two isolates, JZ4 and JZ7, which were further quantitatively characterized demonstrated promising effects for promoting salinity stress tolerance of *A*. *thaliana*. A draft genome of isolate JZ4 has been previously published [66]. Other isolates from Jizan and other locations have also been accomplished [67–72], along with a complete genome sequence of a plant growth promoting bacteria *Enterobacter* sp. SA187 which has been shown to increase the crop yield of alfalfa under saline irrigation and desert conditions [73, 74]. The effect of the bacteria on plant physiology will also be investigated, to clarify the mode of action of the individual isolates. In conclusion, in addition to being a promising tool to understand the mechanisms by which PGPB functions are mediated, bacteria isolated from desert plants also exhibit a promising solution for sustainable agriculture in arid and semi-arid regions such as North Africa and the Middle East [75–78].

## Supporting information

S1 Fig Experimental design of the study. Geographic location of selected pioneer desert plant species **(A)**. Samples used for 16S bacterial community analysis and isolation of culturable bacteria **(B)**. Screening assays of culturable bacterial root endophytes for PGP traits **(C)** and salinity stress tolerance promotion on *Arabidopsis thaliana* **(D)**.
(TIF)

S2 Fig Qualitative analysis of PGP traits and survival in abiotic stresses of endophytic bacterial collection from each plant species from Jizan. P Sol.—calcium phosphate solubilization; Sid.—siderophore production; ZnO, ZnCO_3_ Sol.—zinc oxide/carbonate solubilization; IAA—indole-acetic acid production; NaCl 5%—growth on 5% NaCl; PEG 8,000 20%—growth on 20% PEG 8,000; open circle—negative ability; closed circle—positive ability.
(TIF)

S3 Fig Effects of bacterial inoculation on *A*. *thaliana* plant growth under salinity stress. Representative images taken 16 DAT on ½MS supplemented with 100 mM NaCl. The shapes and symbols displayed below each image for each isolate are used for indicating the PGP traits possessed by the isolate, its ability to tolerate abiotic stresses, the host plant species it was isolated from and the phylum it belongs to. Mock: (bacteria free LB control). P Sol.—calcium phosphate solubilization (red hexagon); Sid.—siderophore production (blue hexagon); ZnO, ZnCO_3_ Sol.—zinc oxide/carbonate solubilization (green/yellow circle); IAA—indole-acetic acid production (purple triangle); NaCl 5%—tolerance to salt stress (black square); PEG 8,000 20%—tolerance to drought stress (white square); *T*. *terrestris* (black, 4 point star), *Z*. *simplex* (black, 7 point star), *P*. *turgidum* (black, 6 point star), *E*. *granulata* (black, 5 point star); Actinobacteria (white, 7 point star); Firmicutes (white, 6 point star); Proteobacteria (white, 5 point star). White bars in photographs correspond to 1 cm.
(TIF)
