# Supplementary figures and images for: Desert plant bacteria reveal host influence and beneficial plant growth properties
Source: PLoS One. 2018 Dec 12;13(12):e0208223. doi: 10.1371/journal.pone.0208223 (PMC6291088; doi:10.1371/journal.pone.0208223)

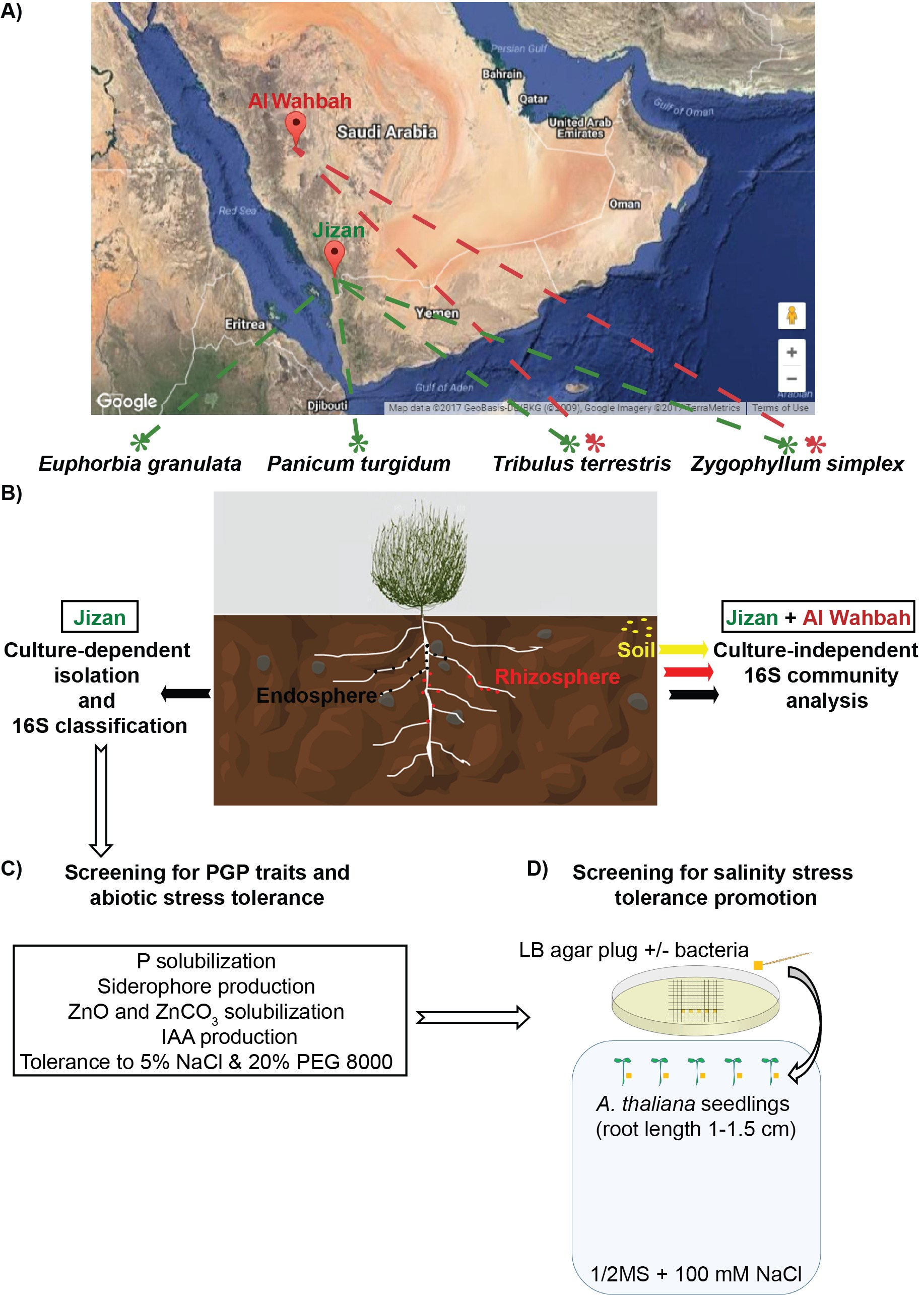

Supplement: S1 Fig — Geographic location of selected pioneer desert plant species (A). Samples used for 16S bacterial community analysis and isolation of culturable bacteria (B). Screening assays of culturable bacterial root endophytes for PGP traits (C) and salinity stress tolerance promotion on Arabidopsis thaliana (D). (TIF) [file pone.0208223.s001.tif]

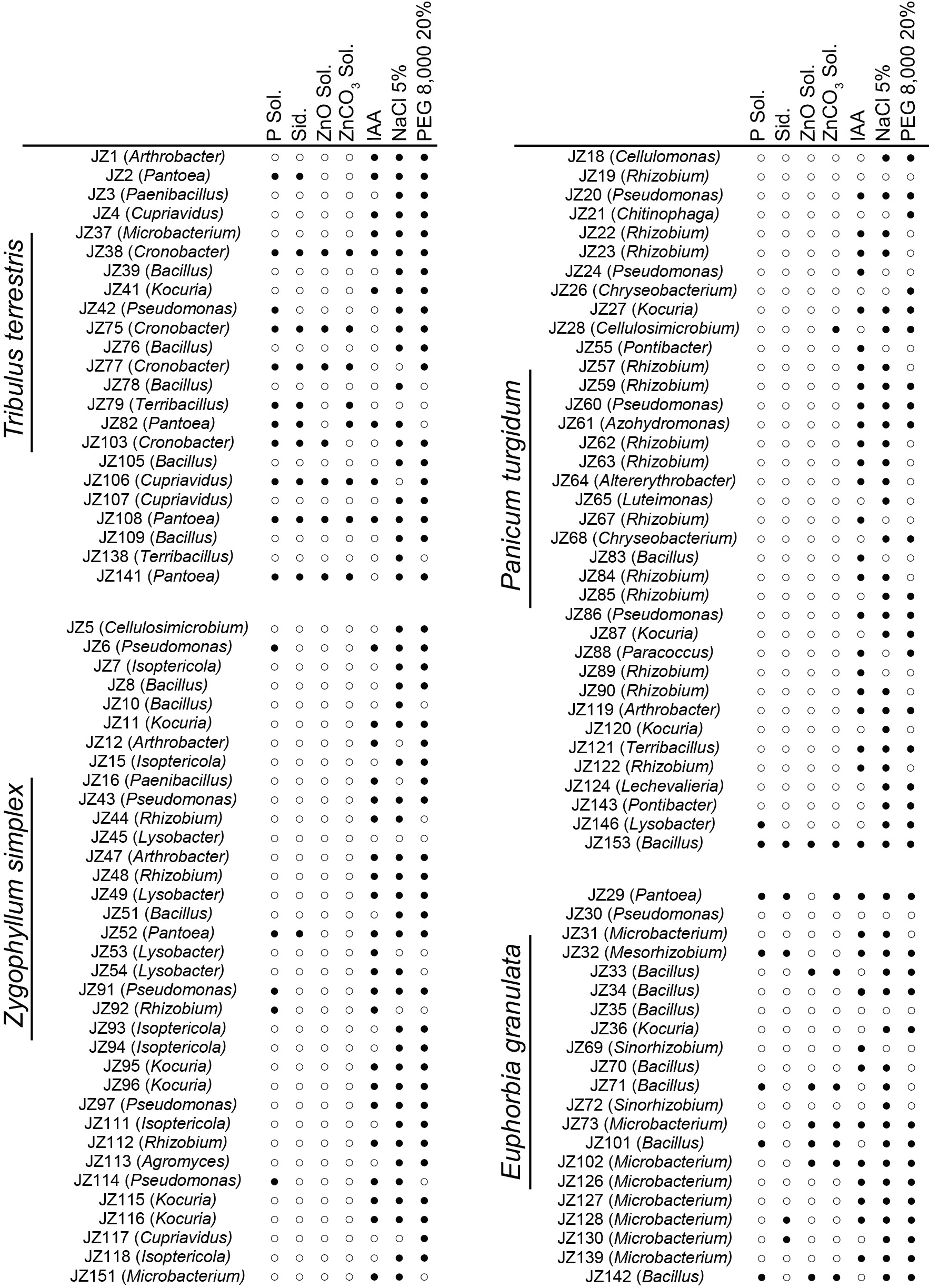

Supplement: S2 Fig — P Sol.—calcium phosphate solubilization; Sid.—siderophore production; ZnO, ZnCO3 Sol.—zinc oxide/carbonate solubilization; IAA—indole-acetic acid production; NaCl 5%—growth on 5% NaCl; PEG 8,000 20%—growth on 20% PEG 8,000; open circle—negative ability; closed circle—positive ability. (TIF) [file pone.0208223.s002.tif]

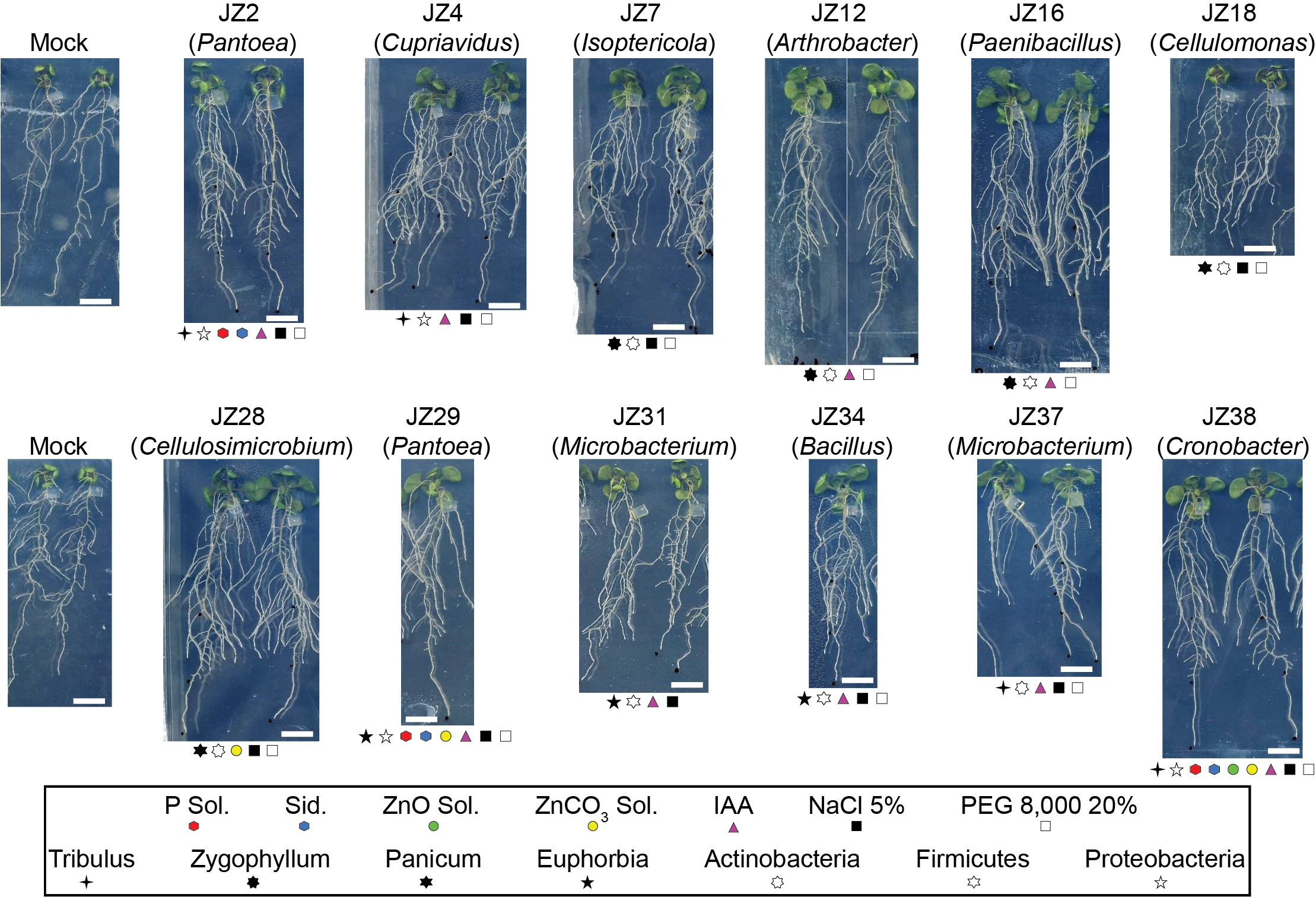

Supplement: S3 Fig — Representative images taken 16 DAT on ½MS supplemented with 100 mM NaCl. The shapes and symbols displayed below each image for each isolate are used for indicating the PGP traits possessed by the isolate, its ability to tolerate abiotic stresses, the host plant species it was isolated from and the phylum it belongs to. Mock: (bacteria free LB control). P Sol.—calcium phosphate solubilization (red hexagon); Sid.—siderophore production (blue hexagon); ZnO, ZnCO3 Sol.—zinc oxide/carbonate solubilization (green/yellow circle); IAA—indole-acetic acid production (purple triangle); NaCl 5%—tolerance to salt stress (black square); PEG 8,000 20%—tolerance to drought stress (white square); T. terrestris (black, 4 point star), Z. simplex (black, 7 point star), P. turgidum (black, 6 point star), E. granulata (black, 5 point star); Actinobacteria (white, 7 point star); Firmicutes (white, 6 point star); Proteobacteria (white, 5 point star). White bars in photographs correspond to 1 cm. (TIF) [file pone.0208223.s003.tif]
